# The reliability and validity of radiographic measurements for determining the three-dimensional position of the talus in varus and valgus osteoarthritic ankles

**DOI:** 10.1007/s00256-012-1421-6

**Published:** 2012-05-19

**Authors:** Tomasz L. Nosewicz, Markus Knupp, Lilianna Bolliger, Beat Hintermann

**Affiliations:** 1 Department of Orthopaedic Surgery & Traumatology, Kantonsspital Liestal, Rheinstrasse 26, 4410 Liestal, Switzerland; 2 Department of Orthopaedic Surgery, Academic Medical Center, Meibergdreef 9, 1105 AZ Amsterdam, The Netherlands

**Keywords:** Ankle osteoarthritis, Talar position, Varus, Valgus, Radiographic measurements, Reliability, Validity

## Abstract

**Objective:**

To assess the most accurate radiographic method to determine talar three-dimensional position in varus and valgus osteoarthritic ankles, we evaluated the reliability and validity of different radiographic measurements.

**Materials and methods:**

Nine radiographic measurements were performed blindly on weight-bearing mortise, sagittal, and horizontal radiographs of 33 varus and 33 valgus feet (63 patients). Intra- and interobserver reliability was determined with the intraclass coefficient (ICC). Discriminant validity of measurements between varus and valgus feet was assessed with effect size (ES). Convergent validity (Pearson’s *r*) was evaluated by correlating measurements to the dichotomized varus and valgus groups. Obtained measurements in both groups were finally compared with each other and with 30 control feet.

**Results:**

Reliability was excellent (ICC > 0.80) in all but two measurements. Whereas frontal plane validity was excellent (ES and *r* > 0.80), horizontal and sagittal measurements showed poor to moderate validity (ES and *r* between 0.00 and 0.60). Four measurements were significantly different among all groups (*p* < 0.05). Talar positional tendency was found towards dorsiflexion or endorotation in the varus group and towards plantarflexion or exorotation in the valgus group. The frontal tibiotalar surface angle, sagittal talocalcaneal inclination angle, and horizontal talometatarsal I angle showed the best reliability, validity, and difference among the groups.

**Conclusion:**

The frontal tibiotalar surface angle, sagittal talocalcaneal inclination angle, and horizontal talometatarsal I angle accurately determine talar three-dimensional radiographic position in weight-bearing varus and valgus osteoarthritic ankles. Careful radiographic evaluation is important, as these deformities affect talar position in all three planes.

## Introduction

Correct radiographic evaluation of complex foot and ankle deformities remains a most challenging issue. This is particularly true for ankle osteoarthritis, where as many as 63% of all patients present with varus or valgus talar misalignment [1]. The multitude of additionally performed procedures during surgery [2–4] further suggests that deformity in varus and valgus ankles is not confined, in many cases, to the frontal plane. Understanding of the deformity in all three dimensions, therefore, remains one of the main problems in skeletal radiology and corrective surgery.

As most new imaging techniques do not allow the foot to be assessed while loaded, standard weight-bearing radiography in foot and ankle abnormalities still remains the mainstay of quantitative evaluation of the deformity and operative planning and assessment. However, in contrast to forefoot abnormalities [5–8], radiographic assessment of hindfoot pathologies is still lacking reliable and validated methods to describe deformities. This is particularly true for the position of the talus in misaligned ankles.

The goal of this study was to determine the most accurate standard radiographic method to describe the three-dimensional position of the talus in weight-bearing varus and valgus osteoarthritic ankles. Hence, the results of different radiographic measurements in varus and valgus deformities were evaluated according to their reliability and validity, and the obtained parameters were compared with each other and with a control group.

## Material and methods

### Patient and control group inclusion

Between 2008 and 2010, 66 ankles from 63 consecutive patients who were treated at our institution for varus or valgus ankle osteoarthritis and who fulfilled the criteria of not having undergone previous arthrodesis, ligament reconstruction, or tendon transfer on the affected foot were assessed (Table 1). The study was approved by the hospital’s internal review board, and informed consent was obtained from all patients in accordance with the World Medical Association’s Declaration of Helsinki.

**Table 1:** Demographics for patients (varus/valgus; 33 feet each) and controls

		Patients	Controls
Number of feet		66	30
Age (years)	Mean ± SD	68 ± 8*	44 ± 12*
	Range	51–85	17–67
Gender (male:female)		39:27	19:11
Side (right:left)		37:29	14:16

*p = 0.00

There were 33 varus and 33 valgus ankles. This sample size was based on the optimal design for reliability studies as described by Walter et al. [9]. Minimum intraclass coefficient (ICC) was set at 0.8, and expected ICC at 0.9. With three measurement replicates in 33 samples per group, a significance level of 0.05 with a power of 80% was obtained [9].

Arthritis was etiologically classified according to Valderrabano et al. [1] into posttraumatic, secondary, and primary arthritis. Posttraumatic arthritis was further subdivided into osseous or ligamentous arthritis. Secondary osteoarthritis consisted of systemic diseases, such as rheumatoid arthritis, or arthritis after longstanding deformity, e.g., cavovarus or planovalgus. If no causative factor could be identified, arthritis was classified as being primary (Table 2).

**Table 2:** Osteoarthritis (OA) etiology

			Varus	Valgus
Etiology (*n*/%)	PT	Sprain	13/39	8/24
		Fracture	14/42	16/49
	Primary OA		2/6	4/12
	Secondary OA		4/12	5/15
Total (*n*/%)			33/100	33/100

*PT* Posttraumatic

To compare the varus and valgus groups with the normal situation, a control group was formed that included 30 skeletally mature individuals who were treated at our institution and had not undergone previous ankle surgery (Haglund disease, 10; contralateral supramalleolar osteotomy, 10; and contralateral fracture, 10; Table 1).

In all three groups, radiographic assessment consisted of a mortise ankle view (foot 15° endorotated), combined with sagittal and horizontal views of the foot and ankle. All radiography was performed with the use of the Philips DigitalDiagnost (Philips Research, Eindhoven, the Netherlands). The settings for the radiation source in the mortise, sagittal, and horizontal views were 5 mAs and 60 kV, 4 mAs and 60 kV, and 3.2 mAs and 57 kV, respectively. In the mortise view, the beam was focused on the ankle joint, directed equidistant between both malleoli; in the sagittal view, on the medial malleolus; and in the horizontal view, on the first cuneiform bone. The beam was inclined parallel to the floor in the mortise and sagittal views, and inclined 15° caudocranial in the horizontal view. The film focus distance in all cases was 120 cm. All radiographs were taken in weight-bearing stance, with the affected foot bearing approximately 50% of the total weight.

In order to standardize imaging and avoid possible rotational deviations, criteria for proper imaging were formulated (disregarding talar malpositioning in the case of varus or valgus). On the mortise view, these were an open tibiotalar and lateral joint space, minor fibiotibial overlap, and position of the fifth phalanx on the same vertical line as the distal tibiofibular joint; on the sagittal view, parallel lined tibial domes, open tibiotalar joint space, and overlap of the distal fibula over the tibia; and on the horizontal view, equal spacing between the second to fifth metatarsal, overlap between the second to fifth metatarsal bases, and an open joint space between the first and second cuneiform bone.

### Measurement protocol

**Fig. 1:**
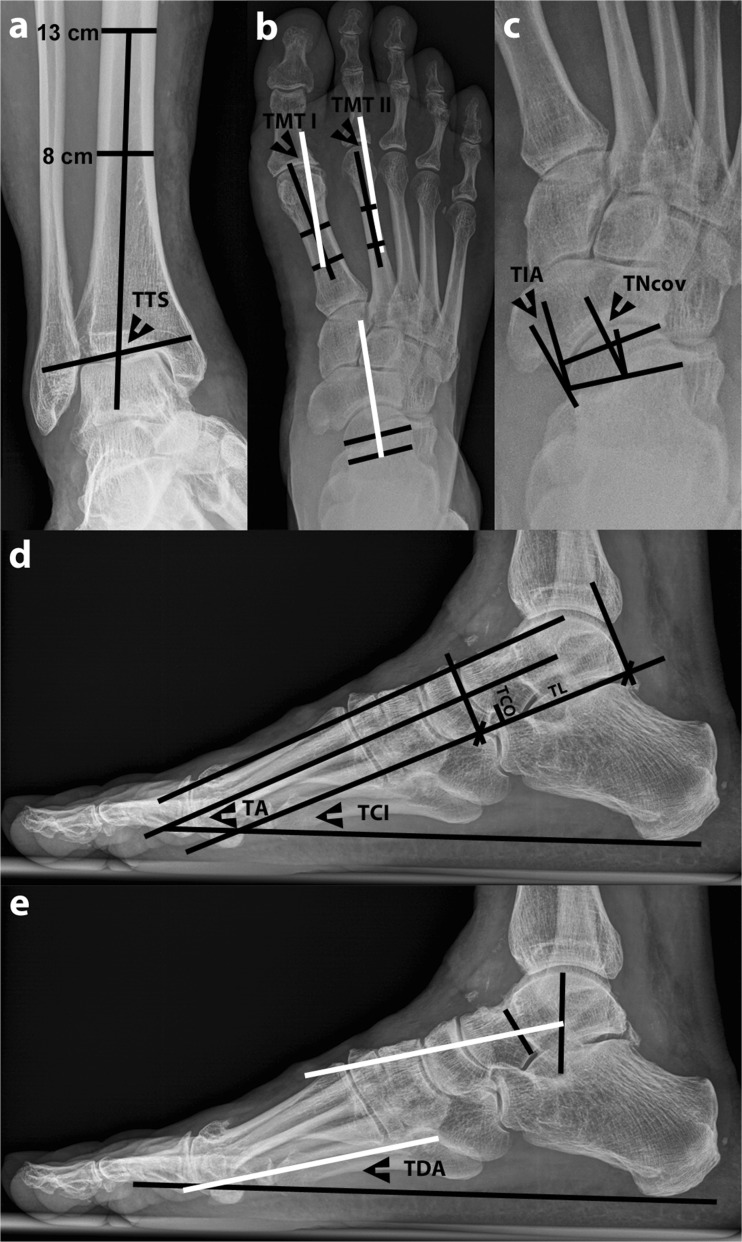
Weight-bearing radiography of a patient with varus ankle osteoarthritis showing the radiographic measurements to determine the three-dimensional position of the talus. The following measurements were performed: **a** Frontal tibiotalar surface angle (TTS): the mid-longitudinal tibial axis was formed by a line bisecting the tibia at 8 and 13 cm above the tibial plafond. **b** Horizontal talometatarsal I and II angles (TMT I and II): all* white lines* run parallel and represent the talar axis. **c** Talonavicular coverage angle (TNcov) and talonavicular incongruency angle (TIA). **d** Talar angle (TA), talocalcaneal inclination angle (TCI), talocalcaneal overlap (TCO), and talar length (TL). **e** Talar declination angle (TDA): both* white lines* run parallel and represent the talar axis. The tarsal index in **d** was determined by using TCI, TCO, and TL

The selected parameters for radiographic assessment are shown in Fig. 1.

In the frontal plane, the tibiotalar surface angle [10] was measured on the medial side. The lower the value for this angle, the more varus talar tilt was present. Conversely, the higher the angular value, the more valgus talar tilt was present.

In the horizontal plane, the talometatarsal I and II angles, talonavicular coverage angle, and talonavicular incongruency angle were determined [5, 6]. The talometatarsal I and II angles were defined as negative when the talar axis was aligned in an abducted position to the metatarsal axis; similarly, the angles were defined as positive when the talar axis was aligned in an adducted position. The talonavicular coverage angle was defined as positive when the talar articular axis was medial from the navicular articular axis and defined as negative when the opposite occurred. Similarly, the talonavicular incongruency angle was defined as positive when the line connecting the lateral talar neck at its most narrow point and the lateral extent of the talar articular surface was positioned medial from the line connecting the latter point with the lateral aspect of the navicular surface; whereas, when the opposite occurred, the angle was defined as negative. In the horizontal plane, the higher the angular values, the more talar endorotation was present, and the lower the angular values, the more talar exorotation was present.

In the sagittal plane, the talar angle [11], talocalcaneal inclination angle [12], tarsal index [12], and the talar declination angle [13] (by using the talar axis as described by Ellis et al. [5]) were determined. The tarsal index as described by Benink [12] was calculated with the following formula: $TI = 100 \times \left( {TCO \div TL} \right) \times \tan \left( {TCI} \right),$ in which TI is the tarsal index, TCO the talocalcaneal overlap, TL the talar length, and TCI the talocalcaneal inclination angle. In the control group, the sagittal talometatarsal I angle [5–7, 14] was additionally measured for comparison to literature. In the sagittal plane, with increasing talar plantarflexion, the angular and index values would increase. Conversely, with increasing talar dorsiflexion, the angular and index values would decrease.

All images from the varus and valgus groups, regardless of plane, were assigned a random number and were independently and blindly measured by three observers (an orthopedic resident, a human movement scientist, and an experienced foot and ankle surgeon). Control group measurements were performed by one observer (the orthopedic resident). Measurements were performed with the digital measuring program Image Access version 4 (Imagic Bildverarbeitung, Glattburg, Switzerland).

### Statistics

Intra- and interobserver reliability was determined by using single measurement, absolute agreement ICC (2,1). To determine interobserver reliability, the mean of interobserver ICCs was taken. Intraobserver reliability was determined by comparing two measurements performed by one observer (the orthopedic resident) at an interval of 6 weeks.

Discriminant validity, indicating whether measurements discriminate between the varus and valgus groups, was assessed by calculating effect size. Convergent validity, indicating whether measurements correlate to a related observation that classifies varus or valgus, was determined by using Pearson’s correlation coefficient *r*. In this study, measurements were correlated to the dichotomized varus or valgus groups as determined on mortise radiographs. The following classification of ICCs, effect size, and Pearson’s *r* was used to assess the degree of reliability and validity: less than 0.20 equaled poor, 0.20–0.40 low, 0.40–0.60 moderate, 0.60–0.80 good, and more than 0.80 excellent [14].

To compare the varus, valgus, and control groups with each other, ANOVA was performed for three-group testing (level of significance *p* < 0.001), followed by post-hoc analysis using the unpaired Student *t*-test and Bonferroni stepdown adjustment for multiplicity testing of measurements [15]. Normal distribution of data was previously evaluated by a Kolmogorov-Smirnov test. Finally, nominal data were compared with the chi-squared test (level of significance *p* < 0.05). All statistical data analysis was performed with the use of SPSS 17.0 (SPSS, Chicago, IL, USA).

## Results

All measurements except the tarsal index and talonavicular incongruency angle showed excellent intra- and interobserver reliability. Whereas validity was excellent for the tibiotalar surface angle, the remaining measurements showed poor to moderate validity (Table 3).

**Table 3:** Reliability and validity results between the varus and valgus groups

Angle	Reliability		Validity	
	ICC		Discriminant	Convergent
	Intra	Inter (mean ± SD)	ES	Pearson’s *r*
Frontal plane				
TTS	0.99	0.98 ± 0.06	0.87	0.86
Sagittal plane				
TCI	0.94	0.90 ± 0.02	0.57	0.58
TDA	0.95	0.87 ± 0.03	0.52	0.54
TI	0.89	0.74 ± 0.16	0.57	0.58
TA	0.94	0.91 ± 0.00	0.33	0.35
Horizontal plane				
TMT I	0.93	0.86 ± 0.02	0.47	−0.45
TMT II	0.93	0.86 ± 0.03	0.38	−0.39
TNcov	0.94	0.87 ± 0.04	0.16	−0.16
TIA	0.78	0.56 ± 0.10	0.10	−0.11

*ICC* intraclass coefficient, *ES* Effect size,* TTS* tibiotalar surface angle,* TCI* talocalcaneal inclination angle, *TDA* talar declination angle,* TI* tarsal index,* TDA* talar declination angle,* TA* talar angle,* TMT I & II* talometatarsal I and II angles,* TNcov* talonavicular coverage angle,* TIA* talonavicular incongruency angle

The degree of reliability and validity was defined as follows: less than 0.20 equaled poor, 0.20–0.40 low, 0.40–0.60 moderate, 0.60–0.80 good, and more than 0.80 excellent

While in the varus group, talar position showed a tendency towards dorsiflexion or endorotation, in the valgus group a tendency towards plantarflexion or exorotation was seen. With the numbers available, a statistically significant difference (*p* < 0.05) between the varus and valgus groups was found in all measurements, except for the talar angle, talonavicular coverage, and incongruency angles (Table 4). While talar position in the varus and valgus groups differed significantly according to the frontal tibiotalar surface angle (*p* < 0.05) in both groups, when compared with controls, in the sagittal and horizontal planes, only selected measurements were significantly different. In both groups, the range was large for all measurements (Table 4). The additionally measured sagittal talometatarsal I angle in the control group was −0.1 ± 6.8°.

**Table 4:** Results per group

Angle	Varus	Valgus	Control
	Mean ± SD (range)	Mean ± SD (range)	Mean ± SD (range)
Frontal plane			
TTS (°)	74.8 ± 7.3 (60.7 to 86.1) ‡,§	100.5 ± 7.5 (88.3 to 123.0) ‡,§	89.0 ± 2.6 (83.7 to 96.4)
Sagittal plane			
TCI (°)	25.4 ± 8.6 (11.4 to 42.2) ‡,§	36.3 ± 7.2 (21.6 to 55.7) ‡,§	30.5 ± 4.5 (19.9 to 37.9)
TDA (°)	17.1 ± 11.7 (0.1 to 52.1) ‡,§	30.1 ± 9.3 (9.9 to 56.5) ‡,§	26.3 ± 6.0 (15.8 to 38.4)
TI	6.0 ± 7.2 (−14.5 to 21.3) ‡,§	20.5 ± 12.8 (7.3 to 72.9) ‡,§	12.2 ± 5.7 (0.0 to 23.0)
TA (°)	24.9 ± 7.8 (11.1 to 42.9)	30.4 ± 7.8 (9.1 to 52.0)	28.4 ± 3.0 (23.2 to 34.3)
Horizontal plane			
TMT I (°)	11.6 ± 11.0 (−6.3 to 31.3) ‡,§	−2.5 ± 15.1 (−42.9 to 44.3) ‡	3.7 ± 7.9 (−13.8 to 17.1)
TMT II (°)	19.9 ± 12.8 (−2.3 to 45.3) ‡,§	7.5 ± 15.2 (−36.2 to 48.3) ‡	12.1 ± 8.6 (−5.3 to 28.4)
TNcov (°)	22.8 ± 15.1 (−10.8 to 56.1)	16.6 ± 22.3 (−41.2 to 58.3)	17.5 ± 9.7 (−6.9 to 34.6)
TIA (°)	−6.0 ± 35.2 (−95.4 to 47.1)	3.4 ± 52.6 (−154.7 to 63.7)	9.0 ± 18.0 (−46.4 to 40.1)

*TTS* Tibiotalar surface angle,* TCI* talocalcaneal inclination angle, *TDA* talar declination angle,* TI* tarsal index,* TDA* talar declination angle,* TA* talar angle,* TMT I & II* talometatarsal I and II angles,* TNcov* talonavicular coverage angle,* TIA* talonavicular incongruency angle

‡ Significant difference between varus and valgus; § significant difference between varus or valgus and controls (post-hoc Student *t*-test and Bonferroni stepdown, *p* < 0.05)

**Fig. 2:**
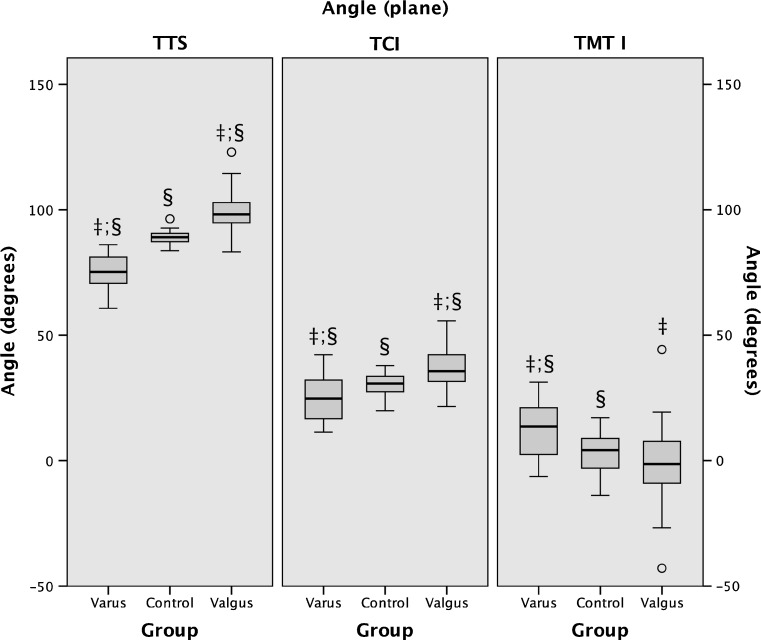
Box plots showing the position of the talus per plane for each group according to the three angles with the best reliability, validity, and difference between groups.* TTS* Frontal tibiotalar surface angle,* TCI* sagittal talocalcaneal inclination angle,* TMT I* horizontal talometatarsal I angle. ‡ Significant difference between varus and valgus; § significant difference between varus or valgus and controls (post-hoc Student *t*-test and Bonferroni stepdown, *p* < 0.05)

Whereas the frontal tibiotalar surface angle, sagittal talocalcaneal inclination angle, and the horizontal talometatarsal I angle showed the best combination of reliability, validity, and difference between groups (Fig. 2), the sagittal talar declination angle and horizontal talometatarsal II angle showed near-identical results.

## Discussion

The position of the talus is crucial in complex hindfoot deformities, such as varus and valgus misaligned ankles. The more the surgeon understands its position, the better he or she may be able to plan and perform operative corrections. The aim of this study was to determine the most accurate method to describe the three-dimensional radiographic position of the talus in varus and valgus osteoarthritic ankles. Other studies, to our knowledge, only reported on forefoot deformities [5–8], hindfoot cavovarus and planovalgus [14], or frontal plane talar tilt [10].

Our results indicate that the frontal tibiotalar surface angle, sagittal talocalcaneal inclination angle, and horizontal talometatarsal I angle are best suited to describe the position of the talus in varus and valgus osteoarthritic ankles in all three planes. These radiographic measurements offered the best combination of reliability and validity and, in general, were significantly different between the groups. In practice, therefore, these angles may be used to accurately quantify deformity, which, in turn, may aid in the preoperative planning and postoperative assessment of reconstructive procedures in varus and valgus osteoarthritic ankles [2–4, 16].

Although nearly identical to our suggested measurements with regard to the obtained results, using the sagittal declination angle and the horizontal talometatarsal II angle would constitute a more cumbersome analysis due to the multiple and smaller reference points used in these angles.

While some measurements were found suitable, other measurements were obviously not. Routine use of these less accurate radiographic measurements to determine the three-dimensional position of the talus in practice may negatively influence deformity assessment. This, in turn, may result in a worse outcome following corrective procedures and is therefore not recommended.

The most unsuitable measurements were found to be the talonavicular coverage angle and talonavicular incongruency angle. Previously, Younger et al. [7] showed that the talonavicular coverage angle did not differentiate between flatfeet and controls. Lower validity in our study might be explained by the navicular following the talus in small talar deviations, making these deviations unaccountable when using the talonavicular coverage angle. Instead, the talometatarsal I angle, although in our study not significantly different between valgus and controls, is independent of navicular landmarks, explaining the higher validity.

The talonavicular incongruency angle, on the other hand, previously was found to reliably differentiate flatfeet from controls [5]. The lower reliability and validity in our study, however, may be explained by debatable and closely positioned reference points. These points, when applied differently, resulted in large angular deviations.

Whereas validity in the frontal tibiotalar surface angle was excellent, the sagittal talocalcaneal inclination angle and horizontal talometatarsal I angle showed moderate validity. However, validity of these latter angles was affected by the wide measurement range and high overlap, reflecting the three-dimensional complexity of the deformities. Thus, despite a distinct sagittal and horizontal talar position tendency in varus and valgus, the talus in both groups could be positioned dorsiflexion or plantarflexed and endorotated or exorotated. In a study in cavovarus and planovalgus feet, Lee et al. [14] found higher discriminant validity in similar measurements due to a more consistent talar position tendency. The authors concluded that these deformities do not occur isolated to the frontal plane. Our results correspond to those of Lee et al. [14], while further showing that the talus in varus and valgus osteoarthritic ankles may ultimately assume a position in each direction in the remaining planes. Adequate radiographic determination of talar position in all three planes is therefore essential prior to performing reconstructive surgery, and in our opinion may be performed by the suggested measurements, despite moderate validity in the sagittal and horizontal plane.

Our suggested measurements allow the position of the talus to be described in all three planes, but there are some limitations. First, we only evaluated the position of the talus in varus and valgus osteoarthritic ankles. In practice, concomitant foot deformities must be viewed separately. The relation of the talus to the distal foot in the sagittal and especially horizontal measurements may, furthermore, be influenced by deformities of the distal foot, i.e. navicular and metatarsals, thereby influencing our measurements. Second, we did not evaluate the influence of talar frontal plane tilt on radiographic landmark positioning. Also, despite using a standardized radiographic imaging technique, any residual rotation deviations may have influenced our final results. Third, we used radiographs to describe the three-dimensional position of the talus. Weight-bearing computed tomography [17], as a potentially more accurate alternative, is, to date, only rarely available and must first prove a higher reliability. Finally, the comparison of varus and valgus feet, with potential radiographic changes following osteoarthritis, with a significantly younger control group may, in some way, have confounded our results. Although our control group was not consecutively or blindly included, control group measurements were, however, consistent with the literature [5, 7, 8, 10, 18], thereby justifying their use in this study.

## Conclusion

The position of the talus in varus and valgus osteoarthritic ankles is not only affected in the frontal plane but also in the sagittal and horizontal planes. Therefore, careful radiographic determination of the three-dimensional position of the talus prior to initiating treatment is important and may best be performed with the frontal tibiotalar surface angle, sagittal talocalcaneal inclination angle, and horizontal talometatarsal I angle.
